# Diagnosis of Chronic Infections with the Gonococcus by the Complement Fixation Test

**Published:** 1919-01

**Authors:** 


### UROLOGY AND DERMATOLOGY

---

**Diagnosis of Chronic Infections with the Gonococcus by the Complement Fixation Test.** By F. B. Bowman and P. D. Taylor. *Bulletin of the Canadian Army Medical Corps*, Aug. 1918.

Concerning the tests carried out at the General Canadian Laboratory, it is evident that in chronic gonorrhea positive results of the discharges by the examination of stained films are very difficult to obtain, chiefly because of the difficulty of obtaining gonococci either intra or extra-cellular.

In the discharges so few gonococci are present, or are so mixed with other bacteria that the Gram-negative diagnosis is almost impossible by freshly stained smears or cultures. In 1916 Müller and Oppenheim used as an antigen simply a suspension of gonococci in salt solution.

Teague and Turrey in 1907 emphasized the importance of using several strains of the gonococcus in preparing an antigen. Schwartz and McNeil emphasized it and stated that reaction never occurred in cases of anterior urethritis.

The authors have always used the anti-sheep hemolytic system. The first antigen prepared by Parke, Davis and Company, made from numerous strains, possessed excellent antigenic qualities. The titration should always be done before the actual fixation test.

The antigen is made isotonic by adding one part of 10 o/o saline to nine parts of the antigen. This is then diluted again 1 : 10 with normal saline and titrated from anti-complementary properties.

When the test proper takes place the patient's serum should be used as soon as possible after taking the specimen. It should be free from cells and clear and heated for one half an hour at 56°C.

From the researches of the C. A. M. C. and others it can be definitely stated that the complement fixation test for gonorrhea is specific and must be relied on to confirm diagnosis, since cultural methods are not practical.

It should be borne in mind (1) that the test is never positive in localized anterior urethritis; (2) that the test is not usually positive before the sixth week of the disease ; (3) that no cross fixation takes place with either syphilitic or tuberculous sera.

It is strongly recommended to keep tested and standardized antigens at hand on account of the difficulty of cultivating strains of gonococci.

